# Dataset for distribution of SIDER2 elements in the *Leishmania major* genome and transcriptome

**DOI:** 10.1016/j.dib.2017.01.001

**Published:** 2017-01-10

**Authors:** Jose M. Requena, Alberto Rastrojo, Esther Garde, Manuel C. López, M. Carmen Thomas, Begoña Aguado

**Affiliations:** a Centro de Biología Molecular Severo Ochoa (CSIC-UAM), Universidad Autónoma de Madrid, 28049 Madrid, Spain; b Instituto de Parasitología y Biomedicina López-Neyra (IPBLN-CSIC), 18016 Granada, Spain

**Keywords:** Leishmania, Repeated sequences, SIDER2, Transcriptome

## Abstract

This paper contains data related to the research article entitled “Genomic cartography and proposal of nomenclature for the repeated, interspersed elements of the *Leishmania major* SIDER2 family and identification of SIDER2-containing transcripts” [1]. SIDER2 elements are repeated sequences, derived from, nowadays, extinct retrotransposons, that populate the genomes of protist of the genera *Leishmania*. This dataset (Supplementary file 1), an inventory of 1100 SIDER2 elements, was generated by surveying the *L. major* complete genome using bioinformatics tools with further manual refinements. In addition to the genomic distribution of these elements (summarized in Fig. 1), this dataset contains information regarding their association with specific transcripts, based on the recently established transcriptome for *L. major*[2].

**Specifications Table**

**Table:** 

Subject area	*Biology*
More specific subject area	*Genome structure and DNA repeats*
Type of data	*Table in MS Excel file, figure*
How data was acquired	*Bioinformatics approaches: BLAST searches on the Leishmania major genome (Friedlin strain) using the server GeneDB (*www.genedb.org*), multiple alignments using Clustal Omega at EBI. RNA-seq analysis tools: Bowtie2, Samtools and custom Python scripts. Also, Python programming was used to extract and format the information in MS Excel sheets*
Data format	*Analyzed*
Experimental factors	*RNA, used for library preparation and Illumina sequencing, was isolated from L. major promastigotes (Friedlin strain, clone V1) cultured at 26 °C in RPMI*
	*medium supplemented with 10% fetal bovine serum, 100 U/ml penicillin G and 0.1 mg/mL streptomycin sulphate. The cells were harvested when the culture density reached 6.1*×*10*^*6*^*cells/mL (mid-logarithmic phase of growth).*
Experimental features	*SIDER2 sequence elements were identified by iterative BLAST searches in the L. major genome database (*http://www.geneDB.org*) using as initial query the consensus sequence previously described*[3]*. The boundaries of the elements belonging to a given subfamily were manually established after visualization of the multiple sequence alignments, which were obtained with the Clustal Omega program [*http://www.ebi.ac.uk/Tools/msa/clustalo/*]. The final coordinates of the elements in the genome were annotated regarding the current version of the L. major genome available at the Sanger Institute (*http://www.sanger.ac.uk/resources/downloads/protozoa/leishmania-major.html*).*
	*Genomic location of SIDER2 elements and their adscription to transcripts were determined after visualization with the IGV browser*[4]*of the location of the SIDER2 elements along the 36 L. major chromosomes and their positioning regarding the L. major transcripts*[2].
	*For determining expression levels of SIDER2-containing transcripts,*
	*RNA-seq reads from a previous work of our group*[2]*were used. Reads were mapped against L. major genome (v6, TritrypDB) using Bowtie2 with the following parameters: --np 0 --n-ceil L,0,0.02 --rdg 0,6 --rfg 0,6 --mp 6,2 --score-min L,0,-0.24. Afterwards, the number of reads mapping to each transcript of the more recent L. major poly A+ transcriptome (*https://www.researchgate.net/publication/302507847_LmjF_MT_v31_reviewed*) were counted using HTSeq*[5]*. Expression levels were calculated using “Transcript per million (TPM)” equations as described by Wagner* et al. [6].
Data source location	*Does not apply*
Data accessibility	*The data are included with this article*

**Value of the data**

• In the genome of the protist *Leishmania*, a remarkable expansion of retroposon-derived, repeated sequences has occurred, originating two large families: SIDER1 and SIDER2 [7]. Here, we are providing a complete list of the repeated sequences belonging to the family SIDER2, including genomic coordinates for a rapid location in the *L. major* (Friedlin strain) genome, which is the reference genome for people working in the field. A figure is also provided for a visual localization of the SIDER2 elements along the 36 chromosomes.
• Based on sequence conservation, SIDER2 elements have been grouped into subfamilies, which were named accordingly to reflect their structural relationships. This analysis has evidenced a conspicuous constraint, regarding chromosomal distribution, in the different subfamilies [1].
• It is included a list of poly-A transcripts containing SIDER2 elements, and their relative expression levels, on the basis of the *L. major* transcriptome, recently established by our group [2].
• This information will be helpful for better understanding the roles played by these degenerate retroprosons in both regulation of gene expression and genome plasticity.

## 1 Data

We are contributing a list of 1100 SIDER2 elements present in the current reference genome of *L. major*. For each element, the following features are provided: genomic coordinates and strand orientation, its grouping to a given subfamily (if any), and the transcript where it is located (if mapped within a poly-A^+^ transcript; otherwise, see below). Giving the putative relevance of SIDER2 sequences in regulating mRNA stability, information related to the orientation (sense or antisense) and location (5′-UTR, CDS, 3′UTR, SL-addition site and/or polyadenylation site) of the element in the SIDER2-containing transcripts has been added. Also, the data show the relative expression levels of the SIDER2-containing transcripts in the promastigote stage of *L. major*. For those elements that do not form part of transcripts, it is indicated their genomic location: intergenic region (IR) or strand-switch regions (either convergent (SSRc) or divergent (SSRd)).

## 2 Experimental design, materials and methods

**Fig. 1:**
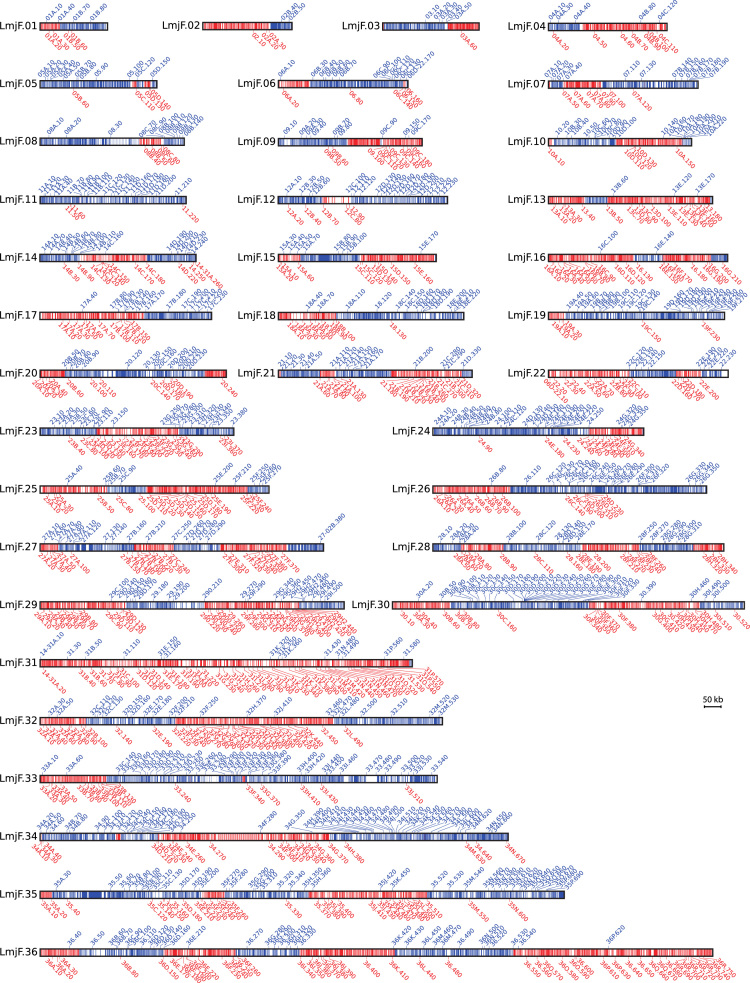
Chromosomal distribution of SIDER2 elements in the 36 chromosomes of *L. major*. For each chromosome, directional gene clusters (DGCs) located on the plus strand are colored in blue whereas those DGCs located on the minus strand are shown in red. Similarly, the names of the SIDER2 elements located on the plus strand are written in blue, above the chromosome drawing, and those located on the minus strand are written in red, below the drawing. The drawings are proportional to the chromosomal sizes.(For interpretation of the references to color in this figure legend, the reader is referred to the web version of this article).

1. The identification of SIDER2 sequence elements in the *L. major* genome was completed in four steps. A consensus sequence, derived from the SIDER2 elements identified in the chromosome 32 of *L. major* [3] was initially used as query for BLAST searches on the genome database of *L. major* (strain Friedlin; version 5.2). Only sequences showing sequence identity ≥ 60% and length ≥100 nucleotides, regarding the consensus sequence, were used to perform an iterative, semi-manual analysis. A new round of BLAST searches in the *L. major* genome with the retrieved sequences was performed to identify additional putative SIDER2-related sequences. Each time a new sequence block was identified, it was used as query for a new BLAST search. In a third step, overlapping sequence blocks were fused to generate the preliminary set of SIDER2 candidates. Finally, an additional round of BLAST searches was done using the sequence for each one of those candidates: if BLAST results showed sequence conservation with more than 30% of the SIDER2 candidates, the element was appointed as *bona fide* SIDER2, otherwise it was discarded.
2. The SIDER2 elements were grouped into subfamilies: a subfamily was defined as the group of SIDER2 elements sharing sequence identity ≥85%. In this step, to define the boundaries of the elements belonging to a given subfamily, the sequences of all the elements were aligned using the multiple sequence alignment program Clustal Omega at EBI. When the size of a given element was clearly different to the average size for the elements of the subfamily, it was cataloged as truncated element. Finally, the genomic coordinates for each one of the SIDER2 were determined after an additional BLAST search on the *L. major* chromosomal contigs (GeneDB database). All this information is provided in Supplementary file 1, and an overall picture of the chromosomal distribution of the SIDE2 elements is shown in Fig. 1.
3. In order to determine the genomic location of the SIDER2 elements and their adscription to transcripts, a GTF file was created using the genomic coordinates of the SIDER2 elements (Supplementary file 1) and visualized in the IGV browser [4] together with the *L. major* transcriptome [2]. According to their location, four categories were established: i) SIDER2 elements located in transcripts derived from currently annotated genes (GeneDB database); ii) SIDER2 elements located in non-annotated transcripts [2] iii) SIDER2 elements located at intergenic regions, between two transcripts with the same transcriptional orientation (IR); iv) SIDER2 elements located between two transcripts with different transcriptional orientation (SSRc or SSRd).
4. In order to calculate the relative expression levels of the SIDER2-containing transcripts, RNA-seq reads from our previous work [2] where used. Reads where mapped against *L. major* reference genome using Bowtie2 [8] with the following parameters: --np 0 --n-ceil L,0,0.02 --rdg 0,6 --rfg 0,6 --mp 6,2 --score-min L,0,-0.24. Reads mapped to each transcript of the more recent *L. major* poly A^+^ transcriptome (available at https://www.researchgate.net/publication/302507847_LmjF_MT_v31_reviewed) were counted with HTSeq [5]. Expressions levels were calculated using “Transcript per million (TPM)” equations as described by Wagner et al. [6]).

## Conflicts of interest

None.

## Appendix A Supplementary material

**Supplementary file 1.** An Excel file containing the 1100 SIDER2 elements identified in the genome of *L. major*. The coordinates for each element are provided, the transcripts in which the elements are embedded (if any) and the relative expression levels (promastigote stage) of the SIDER2-containing transcripts are also included. Abbreviations: 5′UTR and 3′UTR, 5′- and 3′-untranslated regions, respectively; IR, intergenic regions; SSRc and SSRd, convergent and divergent strand-switch regions, respectively.
