# Supplementary material for: Dataset for distribution of SIDER2 elements in the Leishmania major genome and transcriptome
Source: Data Brief. 2017 Jan 10;11:39–43. doi: 10.1016/j.dib.2017.01.001 (PMC5247276; doi:10.1016/j.dib.2017.01.001)
Supplement: Supplementary file 1 — Supplementary material [file mmc1.pdf]

AUTHOR DECLARATION TEMPLATE

We wish to draw the attention of the Editor to the following facts which may be considered as potential conflicts of interest and to significant financial contributions to this work. [OR]
We wish to confirm that there are no known conflicts of interest associated with this publication and there has been no significant financial support for this work that could have influenced its outcome.

We confirm that the manuscript has been read and approved by all named authors and that there are no other persons who satisfied the criteria for authorship but are not listed. We further confirm that the order of authors listed in the manuscript has been approved by all of us.

We confirm that we have given due consideration to the protection of intellectual property associated with this work and that there are no impediments to publication, including the timing of publication, with respect to intellectual property. In so doing we confirm that we have followed the regulations of our institutions concerning intellectual property.

Jose M. Requena
Centro de Biologia Molecular Severo Ochoa
Universidad Autonoma de Madrid
28049 Madrid, Spain
Emails: jm.requena@uam.es
jmrequena@cbm.csic.es

Signed in behalf of all co-authors
